# Primary aldosteronism and obstructive sleep apnea in hypertension: interplay, target organ damage, and clinical management

**DOI:** 10.3389/fendo.2026.1910992

**Published:** 2026-08-03

**Authors:** Lei Yang, Xiang Chen, Lihua Li

**Affiliations:** 1 Department of Gerontology, The First Affiliated Hospital of Dali University, Dali, China; 2 School of Pharmacy, Dali University, Dali, China

**Keywords:** cardiovascular risk, hypertension, obstructive sleep apnea, primary aldosteronism, target organ damage

## Abstract

Primary aldosteronism (PA) and obstructive sleep apnea (OSA) are common but often unrecognized contributors to hypertension. When they coexist, aldosterone excess, fluid redistribution, intermittent hypoxia, sympathetic activation, and shared metabolic risk may increase hypertension-mediated organ damage. This narrative review examines the evidence linking PA and OSA, with emphasis on mechanisms, cardiovascular and renal consequences, diagnostic limitations, and clinical management. Aldosterone-related sodium retention and nocturnal rostral fluid shift may worsen upper-airway obstruction. Conversely, intermittent hypoxia, sleep fragmentation, and sympathetic activation in OSA may alter renin-angiotensin-aldosterone system activity, although OSA does not account for autonomous aldosterone secretion. Both disorders are associated with oxidative stress, inflammation, endothelial dysfunction, cardiac remodeling, and renal injury. Their overlap should be considered in patients with resistant hypertension, hypokalemia, adrenal incidentaloma, clinically suspected OSA, or organ damage not fully explained by measured blood pressure. Management requires independent confirmation of each disorder, subtype-directed PA treatment with adrenalectomy or mineralocorticoid receptor antagonists, and appropriate OSA treatment with positive airway pressure or other individualized measures. Multidisciplinary care may improve blood pressure and intermediate organ outcomes, but prospective studies are needed to determine whether combined treatment reduces long-term cardiovascular, renal, and cerebrovascular events.

## 1 Introduction

Hypertension remains a leading driver of global cardiovascular morbidity and mortality. Among its secondary etiologies, primary aldosteronism (PA) and obstructive sleep apnea (OSA) are prevalent yet frequently underdiagnosed (1). PA is the most common endocrine cause of hypertension and is detected across a broad severity spectrum, including approximately 5-10% of patients with hypertension and higher proportions in resistant hypertension (1, 2). PA is associated with cardiovascular and renal target organ damage (TOD) that exceeds the risk observed in blood pressure-matched essential hypertension (3, 4). OSA adds intermittent hypoxia, sympathetic activation, sleep fragmentation, and intrathoracic pressure changes that further impair vascular and cardiac function (5, 6). Their coexistence is enriched in obesity, metabolic syndrome, and resistant hypertension, although shared risk factors and variable diagnostic definitions complicate causal interpretation (7, 8). This review synthesizes the mechanistic and clinical evidence for PA-OSA overlap, with particular attention to OSA phenotypes, diagnostic uncertainty, target organ injury, and integrated management.

## 2 Epidemiology and clinical characteristics of PA

### 2.1 PA and high-risk populations

PA is a prevalent but systematically under-recognized cause of hypertension. Estimates vary by setting and diagnostic protocol, but PA is generally found in 5-15% of unselected hypertensive cohorts and in approximately 20% of resistant hypertension cohorts (1, 2, 9, 10). Contemporary guidelines support broader case detection, particularly in patients with hypertension, resistant hypertension, hypokalemia, adrenal incidentaloma, or other features that raise the pretest probability of PA (11).

Most patients with PA are normokalemic, so the absence of hypokalemia does not exclude the disorder (2, 12). This heterogeneity reflects the continuum of aldosterone excess and the modifying effects of sodium intake, kidney function, disease duration, and concomitant medication. Aldosterone escape is a related but distinct compensatory phenomenon. During sustained aldosterone-driven sodium retention, pressure natriuresis, natriuretic peptides, renal hemodynamic changes, and tubular adaptations promote spontaneous natriuresis and diuresis, limiting progressive volume expansion (13). Aldosterone escape therefore helps explain partial restoration of fluid balance, but it should not be presented as the sole explanation for normokalemia. The broader PA phenotype, including normokalemic and subclinical disease, supports active screening rather than reliance on potassium status alone (2, 11, 14).

### 2.2 Clinical manifestations and diagnostic process of PA

Clinical PA is heterogeneous. Muscle weakness, fatigue, palpitations, and metabolic alkalosis may occur when hypokalemia is present, but these features are absent in most patients (2, 12). Hypertension, which may be resistant or severe, is the most consistent presentation. Compared with blood pressure-matched essential hypertension, PA is associated with greater cardiovascular and renal risk (3, 4, 15). PA nevertheless remains underdiagnosed and undertreated (15).

The diagnostic pathway includes case detection, biochemical confirmation when required, and subtype classification (11, 16). Screening commonly uses the aldosterone-to-renin ratio (ARR), interpreted with the plasma aldosterone concentration, renin measurement, potassium status, medication exposure, and assay-specific thresholds. Confirmatory testing may include saline infusion or captopril challenge, although guideline-defined exceptions and local protocols should be considered. After biochemical diagnosis, adrenal imaging and adrenal venous sampling are used to distinguish surgically treatable unilateral disease from bilateral disease that is usually managed medically (11, 16).

Diagnosis remains challenging because PA spans mild to overt phenotypes and can overlap with other low-renin forms of hypertension. Liddle syndrome, apparent mineralocorticoid excess, and medication effects can produce superficially similar biochemical or clinical patterns, so interpretation should integrate renin, aldosterone, potassium, clinical context, and confirmatory or genetic evaluation when indicated (11, 16). Differences in assays, thresholds, and confirmatory protocols also contribute to between-center variability (17).

### 2.3 The association of PA with cardiovascular and renal target organ damage

Compared with age- and blood pressure-matched essential hypertension, PA is associated with more cardiovascular and renal complications. Subclinical findings include left ventricular hypertrophy (LVH), increased carotid intima-media thickness (cIMT), and microalbuminuria (3, 4). This excess risk reflects both the hemodynamic effects of sodium retention and direct tissue effects of inappropriate mineralocorticoid receptor (MR) activation. MR activation in cardiomyocytes, fibroblasts, endothelial cells, and podocytes promotes oxidative stress, inflammatory cell recruitment, and fibrotic remodeling (16, 18).

These effects are not explained by blood pressure alone. In the myocardium, aldosterone-related collagen deposition and extracellular matrix expansion increase stiffness and contribute to diastolic dysfunction, atrial fibrillation, and heart failure risk (18, 19). In the kidney, altered arteriolar tone, glomerular hyperfiltration, and direct podocyte injury can precede proteinuria, nephron loss, and chronic kidney disease (3, 20). Delayed diagnosis allows these processes to continue before PA-specific treatment is started.

PA-directed treatment can reverse part of this remodeling, although the magnitude and timing vary with organ damage, disease duration, and treatment adequacy. Adrenalectomy for unilateral disease and adequately dosed mineralocorticoid receptor antagonists (MRAs) can reduce left ventricular mass and albuminuria and improve cardiovascular risk profiles (16, 21). These observations support early diagnosis, but they do not imply that advanced fibrosis or chronic kidney disease is invariably reversible.

## 3 The relationship between OSA and hypertension

### 3.1 Epidemiology and pathophysiological mechanisms of OSA

OSA is common, and its prevalence increases with obesity and age (22). Hypertension occurs frequently in OSA, and OSA is an important contributor to resistant hypertension (22). Recurrent upper-airway obstruction exposes patients to intermittent hypoxia, sleep fragmentation, and negative intrathoracic pressure swings, each of which can raise blood pressure through partly distinct pathways (6).

Intermittent hypoxia and hypercapnia activate chemoreflex pathways and increase sympathetic nervous system (SNS) activity during sleep and wakefulness. The resulting vasoconstriction and cardiac stimulation contribute to sustained blood pressure elevation (23). Repeated hypoxia and reoxygenation also promote oxidative stress, inflammation, reduced nitric oxide bioavailability, endothelial dysfunction, and arterial stiffness (23). Sleep fragmentation can worsen insulin resistance and weight gain, which may further increase both OSA severity and difficulty controlling blood pressure (24).

### 3.2 OSA phenotypes and diagnostic uncertainty beyond the AHI

OSA is not a single physiological or clinical phenotype. Similar apnea-hypopnea index (AHI) values can arise from different combinations of upper-airway collapsibility, ventilatory control instability, arousal threshold, and pharyngeal dilator muscle responsiveness. Patients also differ in age, obesity, sleepiness, insomnia symptoms, rapid eye movement predominance, positional dependence, and cardiometabolic comorbidity (25). This heterogeneity can modify both target organ risk and response to therapy.

Clinical presentation is also variable. Habitual loud or irregular snoring, witnessed apneas, gasping or choking, unrefreshing sleep, nocturia, morning headache, dry mouth, fatigue, and impaired daytime concentration can raise suspicion, but no single symptom is required. Some patients do not report sleepiness or snoring, and OSA can occur at normal body weight. Examination should assess body habitus, neck circumference, upper-airway crowding, tonsillar enlargement, craniofacial structure, and relevant endocrine disorders such as hypothyroidism and acromegaly (26).

The AHI remains central to diagnosis and conventional severity grading, but event counts do not describe the depth or duration of desaturation, the intensity of arousal, or the clinical effect of sleep disruption. AHI can also vary with body weight, sleep position, alcohol or sedative exposure, fluid balance, and night-to-night variability (26). Sleep apnea-specific hypoxic burden captures the cumulative area of event-related oxygen desaturation and has predicted cardiovascular mortality more consistently than AHI in community cohorts (27). Ventilatory burden and arousal burden provide complementary information, although their prognostic associations differ and clinical thresholds are not yet standardized (28). Symptom-cluster studies likewise identify sleepy, insomnia-like, and minimally symptomatic phenotypes with different cardiovascular profiles despite overlapping AHI categories (29).

Questionnaires such as STOP-Bang and the Epworth Sleepiness Scale support risk assessment and symptom measurement, but they cannot establish or exclude OSA. Diagnosis requires objective sleep testing interpreted in the context of a comprehensive sleep and medical evaluation (26, 30, 31). Polysomnography (PSG) remains the reference standard. Home sleep apnea testing (HSAT) is an alternative for uncomplicated adults with symptoms and a high pretest probability of moderate-to-severe OSA, provided that the study is technically adequate and ordered and interpreted within a clinical pathway (30, 31).

HSAT does not measure sleep using electroencephalography and typically reports a respiratory event index based on the recording time. It may therefore underestimate event frequency when wake time is included or when respiratory events cause arousal with little desaturation. A negative, inconclusive, or technically inadequate HSAT should be followed by PSG when clinical suspicion remains (26, 30, 31). PSG is preferred in patients with substantial cardiorespiratory disease, neuromuscular weakness, awake or suspected sleep-related hypoventilation, chronic opioid use, stroke, or severe insomnia. Repeat PSG can be considered when an initial study is negative, but suspicion persists (30).

The 2025 AASM guideline for medically hospitalized adults addresses a separate clinical setting. It advises diagnostic testing after medical stabilization when feasible and recommends a discharge plan that secures timely diagnostic confirmation and management. These inpatient recommendations complement, rather than replace, general adult diagnostic standards (32). In PA-OSA research and routine care, AHI should be interpreted with oxygenation, sleep fragmentation, symptoms, body habitus, and comorbidity rather than used as a complete measure of biological severity (25–31).

### 3.3 The impact of OSA on cardiovascular and renal target organs

OSA can contribute to target organ damage through mechanical, hemodynamic, and neurohumoral pathways. Intermittent hypoxia, sympathetic surges, and inflammation promote arterial stiffness and atherosclerotic risk (22). During an obstructive event, inspiratory effort against a closed airway increases negative intrathoracic pressure, which raises left ventricular transmural pressure and venous return. Repeated pressure loading, together with hypoxemia, can contribute to left ventricular hypertrophy and diastolic dysfunction (33, 34).

Atrial stretch and autonomic instability also create conditions that favor atrial fibrillation (35). OSA is associated with worse outcomes in several cardiovascular populations, but treatment effects vary by outcome, phenotype, and adherence (36–38). CPAP prevents obstructive events during use and lowers blood pressure modestly on average. Evidence for prevention of major cardiovascular events is less consistent, particularly when nightly use is limited (37, 38).

Beyond the cardiovascular system, OSA is associated with renal dysfunction through intermittent hypoxia, sympathetic activation, hemodynamic stress, inflammation, and oxidative injury (39, 40). Repeated sympathetic surges increase systemic and renal vascular tone, promote post-apneic blood pressure rises, and can reduce renal perfusion. Chronic activation may sustain glomerular hypertension and hyperfiltration before nephron loss becomes clinically apparent. These pathways provide a biologically plausible link between OSA, albuminuria, and declining estimated glomerular filtration rate, but obesity, diabetes, and hypertension remain important confounders (39, 41).

**Figure 1:**
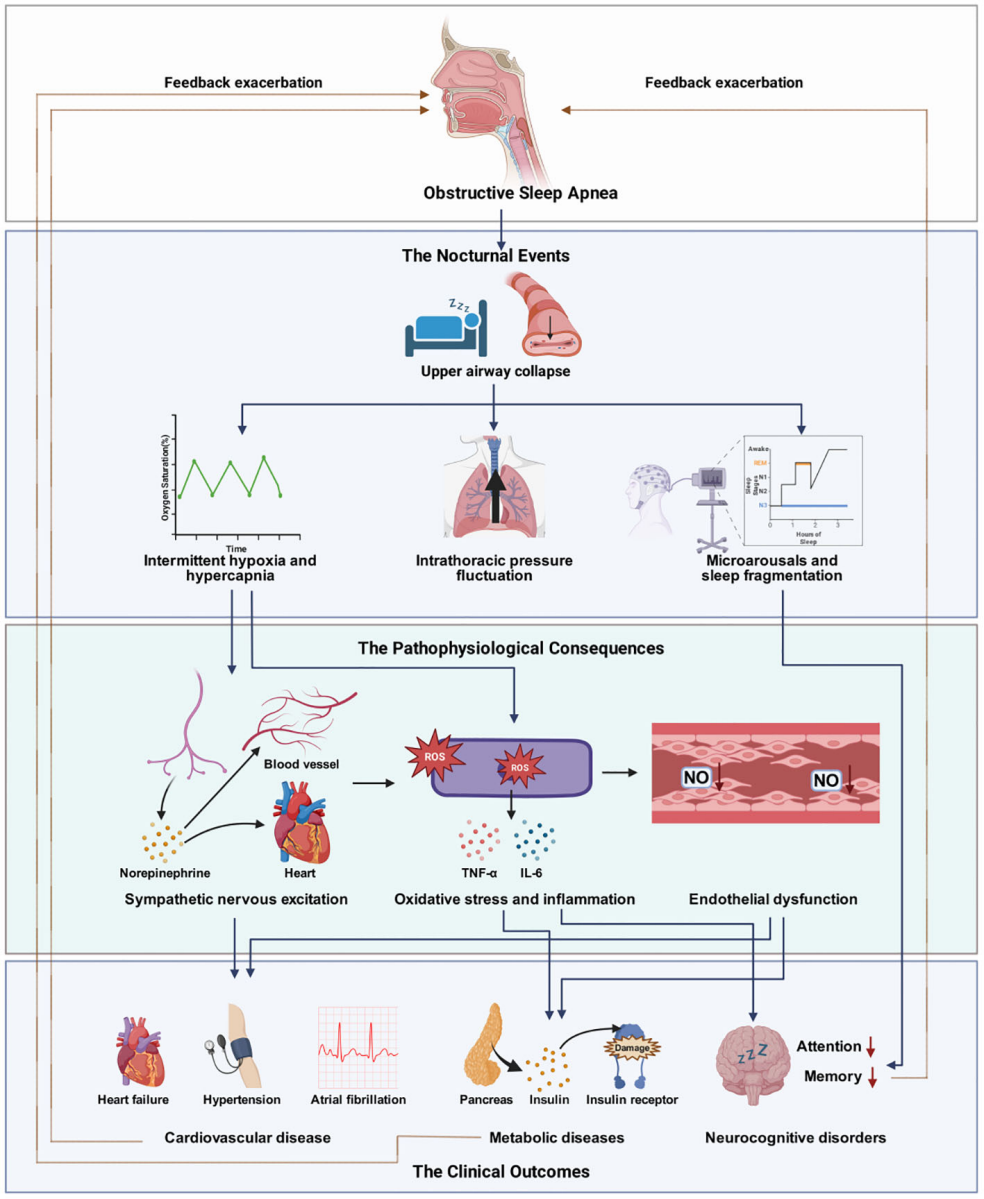
Schematic diagram depicting the pathophysiology and clinical consequences of obstructive sleep apnea. The diagram begins with nocturnal upper-airway collapse, which produces intermittent hypoxia and hypercapnia, intrathoracic pressure changes, and sleep-fragmenting arousals. These events increase sympathetic activity and norepinephrine release, oxidative stress, and inflammatory mediators such as TNF-alpha and IL-6. The resulting endothelial and autonomic dysfunction may contribute to cardiovascular disease, metabolic dysfunction, and impaired attention or memory. OSA, obstructive sleep apnea.

Renal injury may be reinforced by interactions between the sympathetic nervous system and the renin-angiotensin-aldosterone system (RAAS). Intermittent hypoxia and arousal increase sympathetic outflow, whereas renal vasoconstriction and altered sodium handling can further activate neurohumoral pathways (39, 42). In advanced kidney disease, volume overload may worsen OSA through rostral fluid shift (39, 43). CPAP can reduce sympathetic activity and nocturnal hypoxemia, but evidence for durable preservation of kidney function remains mixed (44) (**Figure 1**).

## 4 Comorbid mechanisms of PA and OSA and their impact on target organ damage

### 4.1 Epidemiological link and the “bidirectional” hypothesis

The association between PA and OSA is strongest in cohorts enriched for resistant hypertension, but prevalence estimates vary with referral patterns, obesity, ethnicity, and diagnostic criteria (8, 45). Evidence for the PA-to-OSA direction includes sodium retention, neck fluid accumulation, and improvement in AHI after PA-directed therapy in a small observational cohort (46). Evidence for the OSA-to-aldosterone direction is less uniform. A meta-analysis reported a modest reduction in aldosterone after CPAP, whereas individual studies have produced variable results (47).

These treatment observations require careful interpretation. CPAP splints the upper airway and may reduce OSA-related hypoxemia, sympathetic drive, and secondary RAAS stimulation (37, 46, 47). It does not correct autonomous aldosterone secretion and does not replace PA-directed treatment (11, 16, 46, 47). Conversely, MRAs or adrenalectomy treat aldosterone excess and may reduce fluid-related airway narrowing, but persistent obstructive events still require OSA-directed therapy (11, 37, 46). The available evidence therefore supports reciprocal amplification in susceptible patients, not universal causation in either direction.

### 4.2 Interacting pathways: aldosterone excess, fluid shift, and intermittent hypoxia

Aldosterone excess promotes sodium and water retention and expands extracellular volume. When a patient lies supine, fluid can move from the legs toward the neck, increasing peripharyngeal tissue pressure and upper-airway collapsibility (43, 48). In the opposite direction, recurrent obstruction causes intermittent hypoxia, hypercapnia, arousal, and sympathetic surges. These stresses can alter renin and aldosterone regulation in OSA, especially in resistant hypertension, but they should not be said to create autonomous aldosterone secretion (47, 49, 50).

Atrial natriuretic peptide (ANP) adds an important compensatory link. Negative intrathoracic pressure and transient atrial distension during obstructive events stimulate ANP release. Increased nocturnal ANP promotes natriuresis and diuresis and helps explain the common clinical association between OSA and nocturia. Preventing obstructive events with CPAP reduces nocturnal ANP release and can reduce nocturia (51, 52). In PA, volume expansion activates ANP and other natriuretic pathways that contribute to aldosterone escape (13).

ANP is therefore best viewed as a compensatory signal rather than evidence that OSA causes PA. In patients with both disorders, recurrent nocturnal ANP surges occur against a background of aldosterone-driven sodium retention and altered fluid distribution. This interaction may influence nocturia and overnight volume shifts, but direct studies in well-characterized PA-OSA cohorts remain limited (13, 51, 52).

### 4.3 Obesity, metabolic syndrome, and obesity hypoventilation syndrome

Obesity is a major shared upstream determinant of OSA, resistant hypertension, and metabolic syndrome. Upper-airway fat deposition and reduced lung volume increase airway collapsibility, while visceral adiposity promotes insulin resistance, inflammation, sympathetic activation, and RAAS dysregulation (24). Adipocyte-derived signals, including leptin and other secretagogues, may increase aldosterone production, and mineralocorticoid receptor activation can further worsen adipose inflammation and metabolic dysfunction (53). Thus, PA and OSA may coexist because of shared adiposity-related pathways even when direct bidirectional causation cannot be established.

Obesity is not required for OSA. Male sex, postmenopausal status, craniofacial or pharyngeal crowding, enlarged tonsils or adenoids, hypothyroidism, and acromegaly can increase risk through anatomical or ventilatory mechanisms (26). In a patient with PA, aldosterone-related fluid retention may add an overnight upper-airway load even when generalized adiposity is not prominent (43, 48). This possibility supports assessment of symptomatic or otherwise high-risk non-obese patients, but it does not justify testing every patient with PA (26).

Obesity hypoventilation syndrome (OHS), historically termed Pickwickian syndrome, is defined by obesity and awake alveolar hypercapnia after alternative causes of hypoventilation have been excluded. Most patients have coexisting severe OSA, and reduced chest-wall compliance, restrictive respiratory mechanics, and sustained hypoxemia add cardiopulmonary risk (54). OHS is not a surrogate marker for PA. In an obese patient with OSA or OHS, PA testing should follow hypertension and other established PA indications. Arterial blood gases, serum bicarbonate, and appropriate sleep testing are used to evaluate suspected hypoventilation (11, 30, 54).

## 5 Mechanisms of damage to the cardiac target organs

### 5.1 Aldosterone-mediated myocardial remodeling

Aldosterone also has direct, blood pressure-independent cardiac effects. MR activation in cardiomyocytes and cardiac fibroblasts promotes oxidative stress, macrophage recruitment, profibrotic signaling, extracellular matrix deposition, and myocardial fibrosis (18). Patients with PA therefore have greater LVMI and poorer diastolic function than blood pressure-matched essential hypertension cohorts (4). Fibrotic remodeling can also alter electrical conduction and increase atrial fibrillation risk (19).

### 5.2 OSA-induced myocardial ischemia and sympathetic surge

OSA adds repeated mechanical and neurohumoral stress. Inspiratory effort against an occluded airway increases negative intrathoracic pressure, left ventricular transmural pressure, and venous return. Together with intermittent hypoxia and sympathetic surges, this pressure loading can promote left ventricular hypertrophy (6, 34). Atrial stretch and autonomic instability further increase susceptibility to atrial fibrillation (35).

### 5.3 The combined cardiac phenotype

When PA and OSA coexist, aldosterone-mediated fibrosis may combine with intermittent hypoxia, sympathetic surges, and intrathoracic pressure loading. The overlap can favor left ventricular hypertrophy, diastolic dysfunction, atrial remodeling, and atrial fibrillation (6, 18, 22, 55). These mechanisms are clinically compatible with progression toward heart failure with preserved ejection fraction, although direct comparative outcome studies in rigorously phenotyped PA-OSA cohorts remain limited.

This combined cardiac phenotype should be interpreted as a mechanistic model rather than a quantified multiplicative effect. Blood pressure severity, obesity, age, diabetes, and treatment adherence can confound observed associations. The model supports evaluation and treatment of both disorders when otherwise indicated, together with standard assessment of left ventricular structure, rhythm, and heart failure risk (6, 18, 22, 55).

## 6 Mechanisms of damage to the renal target organs

### 6.1 Complementary aldosterone- and OSA-mediated renal injury

Aldosterone promotes renal injury through hemodynamic stress and direct mineralocorticoid receptor activation in podocytes, vascular cells, and the tubulointerstitium. Oxidative stress, inflammasome signaling, macrophage recruitment, and extracellular matrix deposition contribute to podocyte injury, albuminuria, and progressive fibrosis (20, 56). Early glomerular hyperfiltration can temporarily mask this structural injury.

OSA adds recurrent renal hypoxia and sympathetic vasoconstriction. Post-apneic blood pressure surges and increased renal vascular resistance can reduce renal perfusion, while chronic sympathetic activation, inflammation, and oxidative stress promote glomerular and tubulointerstitial injury (39, 41). The two disorders therefore converge on renal hemodynamic stress and fibrosis through partly distinct pathways.

### 6.2 Combined renal stress and post-treatment renal unmasking

PA and OSA can converge on glomerular hemodynamic stress, hypoxia, inflammation, and fibrosis. Before PA treatment, aldosterone-driven volume expansion and intraglomerular hypertension may cause hyperfiltration that overestimates baseline kidney function. Adrenalectomy or effective MRA therapy can then produce an early decline in estimated glomerular filtration rate as hyperfiltration resolves and the underlying renal reserve becomes apparent (20, 57). This PA-related unmasking should not automatically be labeled acute kidney injury or assumed to be more severe solely because OSA is present. Patients with PA-OSA overlap nevertheless warrant monitoring of creatinine, estimated glomerular filtration rate, potassium, volume status, and blood pressure (11, 20, 57).

## 7 The effects of PA and OSA on the cerebrovascular target organs

### 7.1 Endothelial dysfunction and stroke risk

PA is associated with excess cerebrovascular risk beyond the effect of blood pressure alone (7, 58, 59). Mineralocorticoid receptor activation promotes oxidative stress, vascular inflammation, endothelial dysfunction, arterial stiffness, and small-vessel injury (60). OSA adds intermittent hypoxia, sympathetic surges, and impaired nocturnal blood pressure dipping, which can further stress the cerebral circulation (22, 61).

The coexistence of PA and OSA may identify patients with several converging stroke mechanisms. Direct studies comparing cerebrovascular outcomes in PA alone, OSA alone, and combined disease remain scarce. Current evidence supports control of established vascular risk factors and treatment of each confirmed disorder, while the magnitude of any combined effect remains uncertain (7, 22, 58–61).

### 7.2 Intermittent hypoxia and cognitive decline

Repeated hypoxia-reoxygenation in OSA can impair cerebral vascular regulation and contribute to neuroinflammation (61). Population studies associate sleep-disordered breathing and hypoxemia with cognitive impairment, but causality and treatment responsiveness vary by age, comorbidity, and OSA phenotype (62). Coexisting PA may add vascular risk through hypertension and endothelial injury; however, direct evidence that PA-OSA overlap produces a uniquely amplified cognitive phenotype remains limited.

## 8 Screening strategies for PA and OSA

PA screening should follow current guideline criteria and the clinical pretest probability. Hypertension, resistant hypertension, spontaneous or diuretic-induced hypokalemia, adrenal incidentaloma, and early-onset hypertension or stroke remain important indications (11, 63). OSA can strengthen suspicion when it coexists with hypertension, particularly resistant hypertension, but OSA alone does not establish a high probability of PA. In a multi-ethnic cross-sectional study, PA was present in 8.9% of patients with OSA, but the prevalence fell to 1.5% among those without another PA screening indication (64). Economic modeling supports screening in hypertensive OSA populations rather than unselected normotensive populations (65).

Evaluation in the reverse direction should also be risk based. Obesity is the dominant population-level risk factor for OSA, but normal body weight does not exclude the disorder. A sleep history should ask about loud or irregular snoring, witnessed apnea, gasping or choking, nocturia, unrefreshing sleep, morning headache, dry mouth, sleepiness, fatigue, and impaired daytime concentration. Examination should consider neck circumference, upper-airway crowding, tonsillar enlargement, craniofacial features, and blood pressure (26).

Hypothyroidism and acromegaly can increase OSA risk independently of obesity through upper-airway tissue enlargement, edema, and altered ventilatory control (26). Clinicians should therefore consider objective testing in a non-obese patient with PA when symptoms, resistant hypertension, nocturnal hypoxemia, or otherwise unexplained target organ damage raise suspicion. Questionnaires can assist triage but are not diagnostic; PSG or technically adequate HSAT should be selected according to comorbidity and pretest probability, with PSG after a negative or inconclusive HSAT if suspicion persists (30, 31).

For medically hospitalized adults who screen as high risk, the 2025 AASM inpatient guideline favors linkage to sleep expertise and a discharge plan for timely testing and management. In-hospital screening or limited testing should not be treated as definitive diagnosis when confirmatory evaluation is still required (32).

## 9 Treatment advances in target organ damage related to PA and OSA

### 9.1 Treatment methods for PA and their impact on hypertension and target organ damage

For patients with biochemically confirmed PA, treatment aims to control blood pressure, correct hypokalemia when present, and reduce aldosterone-mediated target organ injury. PA-directed therapy may prevent progression or partially reverse established organ damage, depending on disease duration and baseline injury (11, 16).

Unilateral adrenalectomy is the preferred treatment for suitable patients with lateralized PA. In appropriately selected patients, surgery can achieve biochemical remission and is associated with regression of LVH, lower albuminuria, and improved cardiovascular risk profiles (11).

In bilateral disease or when surgery is not feasible, mineralocorticoid receptor antagonists (MRAs), including spironolactone and eplerenone, are used for long-term treatment. Doses are adjusted according to blood pressure, serum potassium, kidney function, tolerability, and the renin response. Adequate MR blockade improves blood pressure and potassium balance and may reduce aldosterone-related cardiac and renal injury. Observational data suggest lower cardiovascular and renal event rates when renin is no longer suppressed during MRA therapy, although treatment selection and residual confounding must be considered (3, 11).

In routine clinical practice, treatment should prioritize accurate subtype classification, prompt use of an adequately dosed MRA when surgery is not indicated, and monitoring of renal function and serum potassium (11, 16). Aldosterone synthase inhibitors may expand options for resistant phenotypes, but their effects on clinical cardiovascular and renal outcomes in PA require confirmation (66).

### 9.2 Treatment methods for OSA and their impact on hypertension and target organ damage

CPAP remains first-line therapy for moderate-to-severe OSA and for symptomatic patients or those with relevant comorbidities (31, 37). By splinting the upper airway, CPAP prevents obstructive events, reduces nocturnal hypoxemia, and improves sleep continuity. Average blood pressure reductions are modest, but larger effects can occur in resistant hypertension, severe OSA, and patients with good nightly adherence (67, 68). Treatment response should therefore be assessed using adherence, symptoms, nocturnal oxygenation, and blood pressure.

CPAP may also affect cardiovascular structure and function by reducing intermittent hypoxia, sympathetic activation, and abnormal intrathoracic pressure. Studies have reported changes in left ventricular hypertrophy, diastolic function, myocardial strain, inflammatory markers, endothelial function, and arterial stiffness, although designs and effect estimates vary (69–74). A recent synthesis found associations between adequate positive airway pressure use and lower cardiovascular or all-cause mortality, but the evidence differed between randomized and non-randomized studies (70).

Whether CPAP preserves kidney function remains uncertain. By reducing nocturnal hypoxemia and sympathetic activation, CPAP could improve renal hemodynamics and reduce albuminuria (75). Some observational studies and small trials report stable estimated glomerular filtration rate or lower urinary albumin excretion among adherent users, whereas larger randomized trials have not shown consistent long-term renal benefit (76, 77). CPAP should therefore be prescribed for established OSA indications, not as a proven treatment to prevent chronic kidney disease progression.

Adherence often limits CPAP effectiveness (78, 79). Mask discomfort, nasal symptoms, and treatment burden are common barriers. Follow-up should address mask fit, pressure tolerance, nasal symptoms, education, and behavioral support (31). If CPAP is not tolerated, mandibular advancement devices, positional therapy, upper-airway surgery, or hypoglossal nerve stimulation may be considered according to OSA severity, anatomy, and comorbidity (80). Weight reduction, alcohol moderation, smoking cessation, and regular physical activity remain appropriate for most patients with OSA (31, 37, 80).

### 9.3 Clinical effects of comprehensive treatment for hypertensive patients with PA and OSA

When PA and OSA coexist, each disorder should be confirmed and treated according to its own indications. PA-directed therapy reduces aldosterone exposure and sodium retention, whereas OSA treatment reduces obstructive events, hypoxemia, and sleep disruption. Concurrent treatment is reasonable because the physiological targets differ, although direct trials of combined therapy remain limited (11, 37, 81, 82).

Unilateral adrenalectomy is recommended for suitable patients with lateralized PA, whereas long-term MRA therapy is used for bilateral PA or when surgery is not appropriate (11, 83). MRA dosing should account for blood pressure, potassium, kidney function, tolerability, and the renin response. Adequate treatment has been associated with regression of LVH, lower albuminuria, and improved cardiovascular risk profiles (3, 11, 83). In parallel, guideline-directed OSA treatment reduces obstructive events and nocturnal hypoxemia (31, 37).

PA-directed therapy and CPAP are mechanistically complementary, but direct trials comparing combined treatment with either intervention alone are limited. MR blockade or adrenalectomy reduces aldosterone exposure and fluid retention, whereas CPAP reduces obstructive events, hypoxemia, and sympathetic activation (46, 81, 84). Their concurrent use may improve blood pressure control and nocturnal hemodynamics when both disorders are confirmed, but additive effects on left ventricular mass, kidney function, or clinical events require prospective study.

Renal function requires close follow-up when PA and OSA coexist. Before PA treatment, aldosterone-related volume expansion and glomerular hyperfiltration can preserve or raise estimated glomerular filtration rate (eGFR) despite structural injury (20, 57). After adrenalectomy or effective MRA therapy, an early fall in eGFR may reflect correction of hyperfiltration and reveal the underlying renal reserve (20, 57). CPAP reduces nocturnal obstruction and sympathetic activation, but its long-term effect on kidney function remains uncertain (76, 77). Creatinine, eGFR, potassium, volume status, and blood pressure should be monitored when PA-directed and OSA-directed treatments are started (11, 20, 57).

Lifestyle measures remain part of treatment. Weight loss, reduced dietary sodium, a heart-healthy diet, regular aerobic and resistance exercise, and limited alcohol intake can improve blood pressure and metabolic risk (11, 31, 37). Psychological or behavioral support may help patients whose fatigue, sleep disturbance, anxiety, or depression interferes with CPAP or medication use (31, 78).

**Figure 2:**
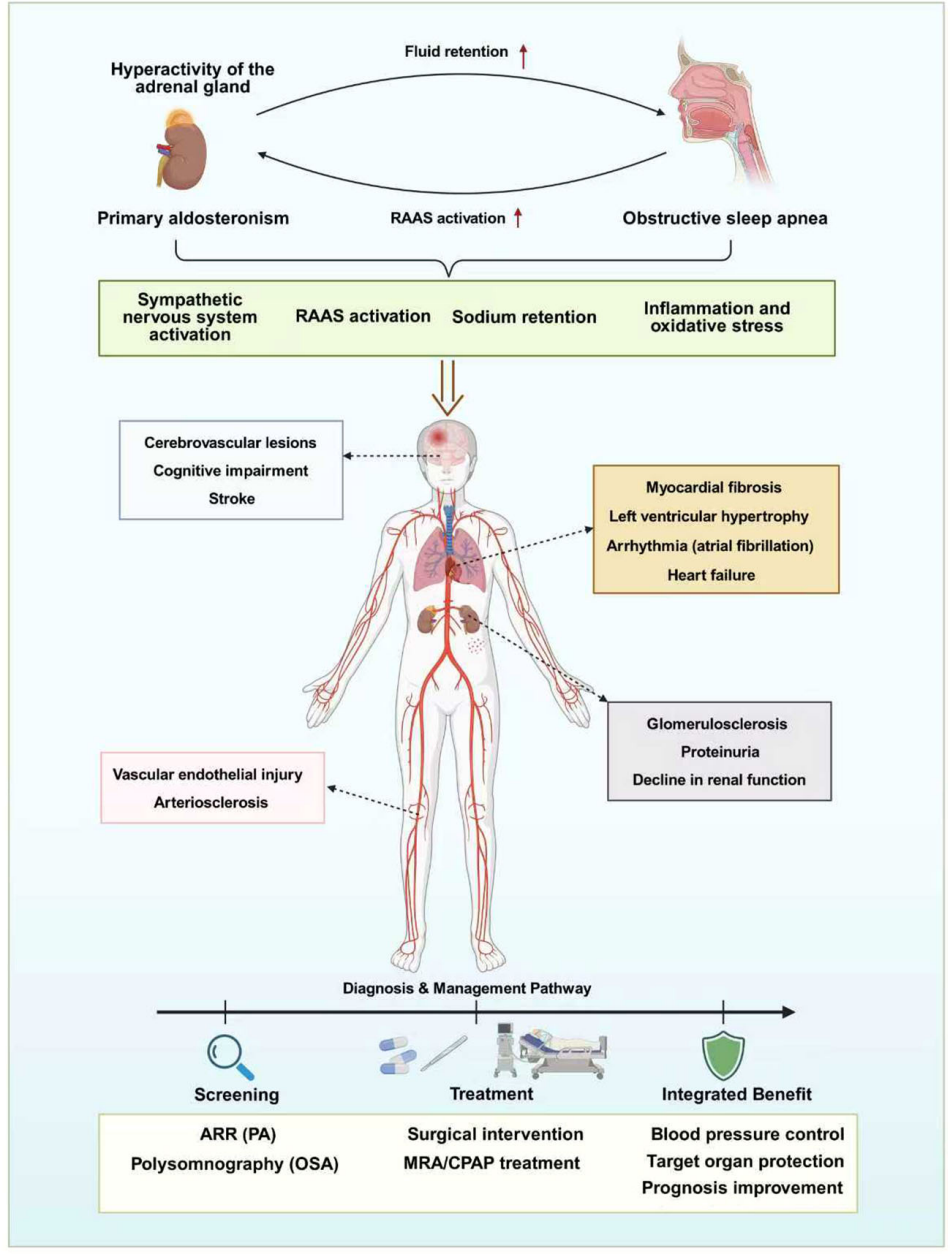
Pathophysiological mechanisms and clinical management pathways link primary aldosteronism (PA), and obstructive sleep apnea (OSA) to hypertension-mediated organ damage and cardiovascular-renal outcomes. The upper section shows pathways that may connect PA and OSA with organ damage. PA causes aldosterone excess and sodium retention. OSA causes intermittent hypoxia, arousal, sympathetic activation, and intrathoracic pressure changes. These processes promote inflammation, oxidative stress, endothelial injury, and damage involving the brain, heart, vasculature, and kidneys. The lower section summarizes clinical evaluation and treatment. PA evaluation uses the aldosterone-to-renin ratio, with confirmation and subtype testing when indicated. OSA evaluation uses PSG or HSAT in appropriate patients. Treatment pairs PA-directed adrenalectomy or MRA therapy with OSA-directed positive airway pressure, weight management, and control of cardiovascular risk. Blood pressure and intermediate organ measures may improve, but long-term outcome data for combined treatment remain limited. ARR, aldosterone-to-renin ratio; CPAP, continuous positive airway pressure; MRA, mineralocorticoid receptor antagonist; OSA, obstructive sleep apnea; PA, primary aldosteronism; RAAS, renin-angiotensin-aldosterone system.

Care should be coordinated among endocrinology, sleep medicine, primary care, and cardiovascular or renal services according to the patient’s organ involvement (11, 31, 37). Treating confirmed PA and OSA addresses different physiological drivers, but the effect of multidisciplinary care on long-term cardiovascular and renal events has not been established. Disease-specific therapy should be accompanied by weight management, sodium reduction, diabetes and lipid care, smoking cessation, and support for treatment adherence (11, 31, 37) (**Figure 2**).

## 10 Conclusion

PA and OSA may coexist through aldosterone excess, fluid redistribution, intermittent hypoxia, sympathetic activation, and shared metabolic risk. Because AHI alone does not describe the full physiological burden of OSA, evaluation should include symptoms, oxygenation, sleep fragmentation, body habitus, comorbidity, and the appropriate objective sleep test. PA testing should follow established biochemical pathways, and OSA testing should follow current diagnostic guidance, including attention to non-obese and hypoventilation phenotypes. Each confirmed disorder requires its own treatment. Combined management may improve blood pressure and intermediate organ measures, but effects on long-term clinical outcomes remain uncertain. Prospective studies with standardized PA diagnostics and multidimensional OSA phenotyping are needed.
